# An analysis of different concepts of “identity” in the heritable genome editing debate

**DOI:** 10.1007/s11019-023-10189-1

**Published:** 2024-01-08

**Authors:** Ying-Qi Liaw

**Affiliations:** Warwick Medical School, University of Warwick, Coventry, England

**Keywords:** Identity, Heritable genome editing, Multi-faceted identity, Genetic identity, Children, Ethics

## Abstract

Human heritable genome editing (HHGE) involves editing the genes of human gametes and/or early human embryos. Whilst ‘identity’ is a key concept underpinning the current HHGE debate, there is a lack of inclusive analysis on different concepts of ‘identity’ which renders the overall debate confusing at times. This paper first contributes to reviewing the existing literature by consolidating how ‘identity’ has been discussed in the HHGE debate. Essentially, the discussion will reveal an ontological and empirical understanding of identity when different types of identity are involved. Here, I discuss genetic, numerical, qualitative and narrative and how each of them is relevant in the HHGE context. Secondly, given the different types of identity, the paper explores how we could navigate these different interpretations of identity in a way that promotes an inclusive and informed discussion between primary stakeholders and the general public in the HHGE debate. Here, I argue for and refine a multi-faceted concept of identity as a suitable framework for discussing the ethical and societal implications of HHGE because it not only could integrate different understandings of identity but also highlight the interconnectedness between these different understandings.

## Introduction

Human heritable genome editing (hereinafter referred to as ‘HHGE’), sometimes also known as human germline editing, involves editing of genes on our sperms or eggs (known as germ cells or gametes) or early human embryos. Potentially, HHGE can be used to prevent the transmission of genetic diseases (NASEM 2017, p. 113). The modifications of genes under HHGE bring heritable effects whereby the edited genes will be passed on to future generations (should the resulting individuals decide to have biological offspring in the future) (NASEM 2017, p. 111). As such, this technological intervention ‘would not only affect individual patients or humans but the human species as a whole’ (de Miguel Beriain 2018, p. 1) and it has been described as one of the ‘disruptive’ technologies (Martin et al. 2020) where its regulation should be aligned with societal needs and values (Ribeiro et al. 2018). It would therefore be necessary for an extensive consideration of the potential ethical, social, medical and regulatory implications of HHGE to continue taking place before going ahead with any clinical application of HHGE. The closing statement of the 2023 International Summit on Human Genome Editing further affirms this as it states that ‘[HHGE] remains unacceptable at this time. Public discussions and policy debates continue and are important for resolving whether this technology should be used. Governance frameworks and ethical principles for the responsible use of [HHGE] are not in place. Necessary safety and efficacy standards have not been met.’

Whilst safety and efficacy issues are important considerations in the HHGE debate, it must be noted that the pragmatic aspects should not be the ultimate guide in the ethical decision-making on HHGE as its application ‘may still contradict ethical values or lead to undesirable societal consequences’ (Almeida and Ranisch 2022, p. 7). Thus, in deciding the circumstances under which HHGE could be reasonably justified, there is a need to continuously engage with the public value-laden concepts such as ‘humanness’, ‘naturalness’ or ‘human diversity’ (Almeida and Ranisch 2022). In light of this, this paper examines a distinct yet seemingly related concept, that is the concept of ‘identity’ in the context of HHGE. The concept of identity is a prominent topic in many reproductive technologies including HHGE where ‘choices of interpretative framing’ could ‘shape the space of sense within which bioethical thinking takes place’, leading to different moral conclusions (Griffiths 2021, p. 14). This paper aims to dissect the different interpretations of ‘identity’ in the literature and argue for a multi-faceted concept of identity that highlights the interconnectedness between these different understandings of ‘identity’ for a more balanced and nuanced debate on HHGE.

This paper takes a multidisciplinary approach in consolidating key perspectives on ‘identity’ pertinent to HHGE, addressing the following research tasks. First, it contributes to reviewing the existing literature by exploring how ‘identity’ has been discussed in the HHGE debate. Essentially, the discussion will reveal an ontological and empirical understanding of identity when different types of identity are involved. Here, I discuss genetic, numerical, qualitative and narrative and how each of them is relevant in the HHGE context. Secondly, given the different types of identity, the paper explores how we could navigate these different interpretations of identity in a way that promotes an inclusive and informed discussion between primary stakeholders including scientists, bioethicists, policymakers and the general public in the HHGE debate. Here, I argue for and refine a multi-faceted concept of identity as a suitable framework for discussing the ethical and societal implications of HHGE because it not only could integrate different understandings of identity but also highlight the interconnectedness between these different understandings.

### Genome editing and its potential impacts on ‘identity’: an overview

The concerns about ‘identity’ are frequently raised when contemplating the possibility of modifying the human genome, particularly in the context of HHGE where changes will be inherited. This can be observed in news media (see e.g. Shekhtman, *The Christian Science Monitor* 2015; Garnham, *The Scotsman* 2016) as well as numerous academic journals (see e.g. Liaw et al. 2021; Griffiths 2021; Hauskeller 2004; Glannon 2001; Chadwick 2001), with seemingly different types of identity involved. As indicated, this paper intends to bring together these different types of identity.

At a broader level, it seems that the identity-related questions of HHGE draw attention to the potential impacts of HHGE on individuals’ personal identity and the wider implications for the identity of the human species. In this regard, it is usually an ontological understanding of identity that is at stake, focusing on the fundamental nature of identity. For instance, the concern of how HHGE may ‘affect the very core of who that person is’ (Garnham, *The Scotsman* 2016) could lead to a range of identity-related discussions touching upon ‘numerical identity’ and/or ‘qualitative identity’ (more details about these different types of identity will be discussed in a later section).

In addition to individuals’ identity, concern also arises in regard to the impacts of HHGE on human beings as a whole. For instance, it is stated that ‘(e)diting the human germline (…) also opens up the possibility of creating an entirely new species. This may threaten our way of life and even our very existence as human beings. (…) what is clear now is that genome editing technologies could potentially change our identity as individuals and as a species in profound and fundamental ways’ (Garnham, *The Scotsman* 2016). The latter envisages a collective identity, that is ‘human identity’, and is aligned with what has been the gist of international human rights instruments in relation to HHGE. For example, the European Convention on Human Rights and Biomedicine 1997 (‘Oviedo Convention’) which bans any genetic intervention that aims to change the genome of the descendants (Article 13), explicitly promotes as its central aim the protection of the ‘dignity and identity of all human beings’ (Article 1). This suggests that, as DeGrazia (2005a, b) also points out, it may be ethically troubling if altering one’s identity would lead to changing the core or essence of being a human being. This human identity is the whole identity that is the core of a multi-faceted concept of identity I will discuss later.

Other than an ontological understanding of identity, there is also an empirical understanding of identity which is most apparent in the renewed focus on identity politics from the disability studies in considering the wider socio-ethical impacts with the potential use of HHGE (Feeney and Rakic 2021; Boardman 2020). In this setting, narrative identity (and perhaps qualitative identity) seems to be at the heart of the discussion, and it is applicable to people with genetic-related conditions as individuals as well as a group. This form of identity not only applies to people with genetic conditions but also applies to people who might be born as a result of HHGE as it draws attention to how identity is lived and experienced (Petersen 2006). This is particularly critical considering that widespread use of HHGE could potentially reduce the prevalence of genetic conditions in the human gene pool in ways that current technologies cannot attain (Boardman 2020, p. 126) thereby reshaping societal perceptions and the prevailing narrative of disability.

A multi-faceted concept of identity could take into account both ontological and empirical understanding of identity where different types of identity such as genetic, numerical, qualitative and narrative identity can be considered not in isolation, recognising their interrelated nature. It therefore provides a framework that encourages a more holistic analysis of the broader implications of HHGE to society from an identity lens.

### Introducing a multi-faceted concept of identity

Before delving into different types of identity in the HHGE context, it is worth first briefly introducing the concept of a multi-faceted identity and what it means in this paper. The idea of a multi-faceted identity I propose here is mainly inspired by Kristin Zeiler’s multi-layered concept of identity in her writing in 2007 where she brings together different concepts of identity in philosophy and argues that genetic and/or genomic changes may but not necessarily define the whole of one’s identity. Arguably, she sees one’s identity as a whole in a numerical sense when she sees ‘whether my whole multi-layered identity is changed to the extent that “I” no longer exist in the sense that “I” have become a different person’ as central to the question relating to germline editing (Zeiler 2007, p. 31). It is however unclear to what extent a change to this ‘whole identity’ would become problematic in the context of HHGE. According to Zeiler (2007), while genetics or genomics may affect certain aspects of ‘identity’, the multi-layered nature of identity highlights that different layers hold varying importance at different times. This paper brings the idea forward and further refines the idea by consolidating both ontological and empirical conceptions of identity as a framework for HHGE public debate. Hence, while narrative identity has not been considered and included (at least not explicitly) in Zeiler’s writing, as I shall show it is significant in mine.

What also differs from Zeiler’s in this paper is the acknowledgement of the *interactions* between these notions of identity. The main idea behind the multi-faceted concept of identity as advocated in this paper is not only an awareness of the different concepts of identity (e.g. genetic identity as one facet of one’s whole ‘identity’; narrative identity as another facet) but also to acknowledge, simultaneously, the interactions between these different types of identity. The implication derived from this understanding is that since ‘identity as a whole’ (that is, as my take in this paper, human identity) is seen as having different dimensions, affecting one facet does not necessarily constitute a change of the ‘whole (human) identity’ even though a changed facet may also influence another facet. Not only that, the change to one facet may be more remarkable at one stage of life and may connote different significance to the individual at different stages of life. A multi-faceted concept of identity taken as a framework in discussion could then answer many identity-related questions in HHGE, ensuring that the debate is done inclusively.

In what follows, I will discuss four different types of identity that are most relevant in the context of HHGE: genetic, numerical, qualitative and narrative identity, each directs us to different ethical considerations. Understanding these distinct identities is necessary to contemplate how a multi-faceted concept of identity can act as a framework for the HHGE debate.

## Different types of identity in the genome editing context

### Genetic identity

It is important to first clarify what the term ‘genetic identity’ might entail since this paper focuses on genome editing where considerations of ‘genetic identity’ are inevitable. As with ‘identity’, the term ‘genetic identity’ itself has various interpretations within different contexts (Goekoop et al. 2020). From a literal scientific or biological perspective, the term ‘genetic identity’ entails the structural makeup, functions or roles of the genes (Salvi 2001, p. 536). Zeiler (2007) further distinguishes between ‘genetic identity’ and ‘genomic identity’ based on the distinction between ‘genes’ and ‘genomes’. Humans have two types of genomes: the nuclear genome, inherited both paternally and maternally, and the mitochondrial genome, which is inherited only maternally (Rosenberg and Rosenberg 2012, pp. 96 & 384). The human genomes are made up of genes. Genes are made up of deoxyribonucleic acid, commonly known as DNA. While ‘genome’ and ‘gene’ connote different meanings scientifically, they are often used interchangeably in the literature concerning genetic engineering.^1^ Nonetheless, understood from this scientific viewpoint, ‘genetic identity’ then focuses on the role carried out by the genes (Salvi 2001, p. 536) whilst ‘genomic identity’ relates to the whole or entire set of genetic information from both nuclear and mitochondrial genomes (Zeiler 2007,p. 28). Arguably, any genetic modification directly changes *genetic* identity (Scully 2017, p. 39) but its impact on *genomic* identity remains uncertain.

The ‘gene-genome’ distinction is also implicitly suggested by the wording used in the Oviedo Convention. As indicated, the Oviedo Convention aims to protect the ‘genome’ of the descendants. The word ‘genome’ was chosen deliberately by the Working Party with the reason that such a word is more ‘comprehensive in meaning’ compared to phrases such as ‘genetic constitution’ and ‘genetic characteristics’ and may cover ‘those parts of the genome without any known specific function’ (Steering Committee on Bioethics 2000, p. 68). This indicates that there are many other parts of ‘genes’ within the ‘genome’. The literal scientific understanding of ‘genetic identity’, based on the distinction between ‘genes’ and ‘genomes’ creates a particular loophole within Article 13 of the Oviedo Convention. Although Article 13 prohibits any genetic modification aimed at changing the *genome* of the descendants, it remains unclear whether a change in ‘genetic identity’ in its literal sense would necessarily connote a change in ‘genomic identity’ as well.

What further complicates the matter is that genetic identity and genomic identity can be understood from an individualistic or collective sense. For instance, each of us can have a (different) genomic identity, that is our whole set of genomes, making each of us unique^2^; or if understood collectively, humans can also have a genomic identity in the sense that we as a species have a complete set of genomic information which is different from, say, the genomic identity from another species (e.g. chimpanzees) (Varki et al 2008; Sholtis and Noonan 2010). Hence, there is a need to take into account these viewpoints to clarify the claim that HHGE will (or will not) alter the ‘identity’ of the resulting individual.

Looking at the international efforts to protect the integrity of the human species (Division of the Ethics of Science and Technology of UNESCO 1999), protecting ‘genetic identity’ has arguably meant ‘genomic identity’ in the collective sense as in the genomes of the whole human species (Liaw et al. 2021, p. 8). A relevant ethical question to be considered in this instance is, therefore, how much genetic intervention could be done to the individual to the extent that they would still remain within the human species and retain ‘human identity’. Within this notion where genetic changes are tied to our sense of being human, an ontological understanding of ‘identity’ might help move the debate forward, e.g. to explore what it means to be a human and what consequences might arise from significant modifications to our genetic makeup. This helps us consider to what extent we as a society could tolerate such changes in human identity.

### Numerical identity

There are two possible understandings of numerical identity that are at stake in the current context of HHGE and which would influence the ultimate claim of whether there is a change in the (numerical) identity of the eventual individual born as a result of the intervention. As I will show here, both understandings of numerical identity serve different regulatory and socio-ethical relevance in the HHGE debate.

#### Identity-over-possible-worlds

First, the so-called ‘identity-over-possible-worlds’ (see Mackie and Jago 2022 for a more detailed discussion on this concept) is at stake when the time-dependence view–that one’s existence depends on the time and circumstances of one’s conception–is adopted. This relates closely to the non-identity problem famously coined by Derek Parfit which in its simplest terms, indicates that we may face difficulty in justifying why we opt for or against the use of certain technology for human reproduction because, in either choice, it is unlikely that we cause any ‘harm’ to the resulting child because the child would not have been born at all without that technology (Parfit 1984, p. 359). This suggests that in a non-identity case, the actions are not ‘person-affecting’ (hence, actions are considered ‘impersonal’) because it does not affect specific individuals, but they change which individuals will exist in the future (Feeney and Rakic 2021; Doolabh et al. 2019).

In the first instance, the application of HHGE appears to be a non-identity case because the decision to use or not use HHGE would affect the existence of a particular individual (Zuradzki 2008; Holm 2019; Rulli 2019; Alonso and Savulescu 2021). For instance, Holm (2019) explains, ‘In a context where gene editing is available to them, prospective parents will, with their clinicians, plan the IVF + gene editing, and it is very unlikely that this will happen at exactly the same time and in exactly the same way as it would have happened if gene editing had not been available, i.e., it is highly unlikely that exactly the same ova will be retrieved and fertilised by exactly the same spermatozoon.’ In a similar vein, Alonso and Savulescu (2021) observed that gene editing which involves IVF was ‘a necessary condition’ of Lulu and Nana’s (a genetically modified twin done prematurely by scientist He Jiankui) existence.

Some scholars however have distinguished whether or not it is a non-identity case depending on whether the procedure is done on the human gametes or embryos. The argument goes: editing the human *gametes* may affect who will be created eventually given that if there is any difference in the timing or other factors (e.g. the delay of time due to genome editing procedure), there could be another sperm that eventually fertilised the egg and thus another individual would have come into existence instead; but this is not the case if the genetic intervention is done on the *embryos* when the fertilisation is complete (Omerbasic 2018; Wrigley et al. 2015).^3^

There are important implications on whether the non-identity problem is applicable in the context of HHGE. If HHGE is considered a non-identity case, defining ‘harm’ or ‘unsafety’ for future individuals becomes complex, as the use of HHGE might be the only way they can come into existence. Holm (2019, p. 106) argues that the non-identity problem suggests that almost no use of genome modification in reproduction can be deemed unsafe unless it creates a life that is so miserable it is not worth living.^4^ Cohen (2011), Parker (2005), and Harris (2000) express similar views, challenging whether regulating reproductive technology based on the best interests or welfare of the child is feasible because, according to the non-identity problem, restricting such technology cannot harm the specific children born through it since they would not otherwise exist. In other words, this view suggests that HHGE might not harm any children because the (same identical) children would not exist without technological intervention. Addressing the non-identity problem's implications for HHGE and understanding how future children's interests can be appropriately considered within this framework should then be prioritised in future research.

Although the non-identity problem is often associated with the field of philosophy (see e.g. Feinberg 1986, p. 158), the non-identity debate need not be confined to the realm of philosophers. In fact, the complexity of non-identity also presents an opportunity for more empirical research focusing on public’s perspectives regarding the non-identity problem and its applicability to HHGE. It would be valuable to investigate the significance of non-identity in shaping the lay public’s and stakeholders’ moral decision-making in the face of HHGE. Currently, to the best knowledge of mine at the time of writing, there appears to be very limited data on this with only one known study by Doolabh et al. 2019, focusing on the public’s view on the weight of the non-identity problem in the field of public health. Similar research should be encouraged in the context of HHGE for further insights into the overall governance of HHGE.

On the other hand, there has also been a renewed debate, focusing on distinguishing HHGE from genetic selection in which the former might not be a non-identity case as it does not involve destroying or discarding certain embryos with disabling traits (Feeney and Rakic 2021; de Miguel Beriain 2020, p. 241). In this sense, it has been suggested that HHGE creates a fresh perspective for ethical consideration in that editing human embryos before birth with the purpose of ‘removing disabling traits’ could bring person-affecting benefits – benefiting the otherwise ‘disabled’ embryos (de Miguel Beriain 2020, p. 241). Nonetheless, this reframing of the HHGE debate also sparked significant challenges, particularly from the disability studies which raise concerns that the employment of HHGE to eliminate disabling traits might convey certain societal messages that are contrary to what people living with disabilities really experience in daily life (Boardman 2020, p. 126). In this regard, it is the narrative and qualitative identity that might be at stake (discussed more below).

#### Identity-over-time

Apart from the ‘identity-over-possible-worlds’, another understanding of ‘numerical identity’ is the ‘identity-over-time’ which focuses on the persistence issue – whether the subject continues to exist over time despite changes (Zeiler 2007; DeGrazia 2005a, b,p. 264). A simplistic example of this would be to think about a kidney transplant patient who is likely to remain numerically the same despite a different kidney. A related question in the context of HHGE is how much change can take place via genetic intervention done prior to birth, before changing the ‘identity’ of the individual. The existing ethical discussion on mitochondrial replacement technique (MRT) helps illustrate different approaches to answer this question. First, it has been argued the application of the MRT may change the numerical identity of the resulting child compared to the child who would have been born without the application (in the sense of improving his/her qualitative identity significantly). The reasoning behind this is that the life trajectory of a child born via MRT could diverge so significantly from the child who would otherwise be born with a mitochondrial disease without intervention that they become two distinct individuals (Nuffield 2012, para 4.18). Juth (2016) seems to share a similar stance when he suggests that some germline modification could indeed bring drastic effects to the extent that the individual has become qualitatively different that he ceases to exist. This ground indicates that changes in qualitative identity can indeed affect numerical identity. Following Juth’s reasoning, it is arguable that HHGE intended for the purpose of preventing rare genetic disease may have significant qualitative-identity-affecting, and thus affecting numerical identity.

Second, whether or not there is an identity change has been argued based on the continuity of a biological process. For instance, Liao (2017, pp. 22–25) argues that ‘a new and numerically distinct individual’ will be created in the application of MRT whenever there is a disruption in the cellular or organismic continuity of the original eggs or early embryos. This happens, as Liao argues, when the original egg or early embryo is deprived of its function to regulate and coordinate various processes – this is where the nucleus of the egg (or nucleus of the early embryo) has been removed for the procedure (Liao 2017, pp. 22–25). This reasoning would yield a different conclusion when applied in the case of HHGE. Liao argues that genetic modification conducted on early embryos does not render the creation of a new and distinct individual based on the idea that modifying (instead of removing) components such as the nucleus within the embryo does not disrupt cellular or organism continuity since the cellular function remains untouched (Liao 2017, p. 25). Following this view, it may be suggested that HHGE might not affect numerical identity. Also, considering the epigenetics approach introduced by Boniolo and Testa (2012, pp. 285 & 289), the persistence of a living being over time relies on the continuity of epigenetics^5^ that starts from the zygotic (or embryonic) stage. Accordingly, as long as the process of epigenetics continues to take place, one is considered to be numerically the same. Following this approach, it is arguable that as long as there are ongoing epigenetics processes along with embryonic development, then one’s numerical identity remains unchanged.

The above discussion shows that there might be different grounds as to what makes one persist through change leading to varied conclusions regarding the presence or absence of a change in numerical identity. Nonetheless, amidst these different approaches^6^ and regardless of the specific approach undertaken, there is at least one particular view on numerical identity that remains relevant and critical in the HHGE debate. The central fear with the use of HHGE which concerns whether or not the technology would fundamentally change ‘human identity’ (see e.g. Shekhtman, *The Christian Science Monitor* 2015; DeGrazia 2005a, b) relates to a numerical understanding of identity (identity-over-time). This discussion should be encouraged in the public debate to facilitate a deeper understanding of what it means to maintain this human identity in the face of disruptive technologies such as HHGE and as van Beers (2020, p. 29) raises, what this means to human rights discourse.

Additionally, it must be stressed that a child’s existence or a child’s (numerical) identity only attains social significance once he/she is born. This social significance is facilitated through identity formation, which is closely related to other kinds of ‘identity’ including qualitative and narrative identity (detailed later). Therefore, I conclude that while an understanding of numerical identity is needed to grasp the overall claim of whether HHGE is identity-affecting, it alone is insufficient to provide a comprehensive evaluation of the ethical acceptability of HHGE for its clinical use in reproductive purposes. While a multi-faceted concept of identity may not be able to solve the issue of whether HHGE is a non-identity case or the moral weight that should be given to non-identity cases, it could ensure inclusion and more informed discussion.

### Qualitative identity

Qualitative identity focuses on the aspects of one being constant to oneself and uniquely recognisable as the same individual (Sollberger 2013, p. 3). Two individuals having the same qualitative identity indicate that they share similar attributes or traits (Noonan and Curtis 2022). One simple example would be to think about cloning: in principle, clone A and clone B are qualitatively identical albeit numerically different to each other.

In the context of HHGE, qualitative identity becomes relevant when we consider how genome modification of human embryos or gametes might impact the resulting individual’s traits and characteristics. Bredenoord and others (2011) suggest that the qualitative identity of the future individual is likely to have changed in the course of modification of the mitochondrial genome because one without a mitochondrial disease will have a ‘different life experience, a different biography and perhaps also a different character’. This line of thinking points to the *effects* of certain interventions in the change of qualitative identity where it can similarly apply in the context of HHGE. Particularly where HHGE is used for the purpose of preventing transmission of certain genetic disorders, it is arguable that the qualitative identity of the resulting individual will be changed after the modification because he/she may have a different kind of life should he/she be born with a serious genetic disorder (without the use of HHGE). This understanding inevitably also links to a narrative sense of identity which will be further elaborated shortly.

Qualitative identity may carry less moral weight in deciding whether a procedure should be legitimate (Nuffield 2012, para 4.11) if it is taken by itself as a singular concept. However, it adds nuance to the overall HHGE discussion within a multi-faceted concept of identity that recognises its interconnection with other types of identity. A question worth exploring is whether a change in qualitative identity is likely to bring an adverse impact on the resulting individual (Nuffield 2012, para 4.12). This could advance the debate on HHGE by facilitating further reflection in the following way: suppose HHGE can affect certain qualitative characteristics and thus the life experiences of the resulting individuals, in that case, it may be argued that the technology should only be used in a way that does not bring adverse effects to the individuals that would eventually be born, or at least to ensure that the individual will have an ‘acceptable’ life experience (Green 1997). Discussion should then focus on what these adverse effects could be and/or what this ‘acceptable’ life experience means in the context of HHGE. This essentially links to other aspects of identity, including narrative and as discussed above, numerical identity.

The link between narrative identity and qualitative identity becomes relevant in understanding and advocating for the rights and experiences of individuals with genetic-related conditions or disabilities. It recognises and highlights the diversity and uniqueness of people with disabilities whose lived experiences (their narratives) could help us better understand the broader implications of the use of HHGE. I now turn to narrative identity.

### Narrative identity

The narrative approach to identity (or simply ‘narrative identity’) has gained wide attention across different disciplines, including social science and psychology. In its simplest form, narrative identity can be understood as storytelling where it includes stories conveyed by ‘ourselves to ourselves, ourselves to others and others to us’ (Scully 2017). Since one’s narratives can be formulated based not only on one’s own interpretation (or in Nuffield’s term ‘self-conception’) (Nuffield 2012, para 4.7) but also on others’ understanding of ourselves (what Nuffield calls ‘intersubjective personal identity’), it is rightly claimed that narrative identity is relational (Postan 2017). This understanding also highlights that there are many factors, both from internal and external sources, that can contribute to the formation of one’s narrative identity.

Narrative identity plays a remarkable, but often neglected, role in the context of HHGE as it interconnects with life experiences, genetic makeup and quality of life. As I will show in this section, these elements contribute to the construction and understanding of an individual’s personal narrative as well as the narrative as a group. The awareness of the role of narrative identity and its place within a multi-faceted concept of identity is necessary for a more nuanced and balanced discussion on HHGE, particularly on the broader implications of this technology.

How biotechnologies could impact one’s narrative identity has been discussed in the MRT context. Nuffield, for example, explains that the use of MRT may affect one’s self-conception in the sense that first, being born via MRT may also contribute to one’s self-conception (for instance, as being a product of donor-assisted reproduction) (Nuffield 2012, para 4.10) and secondly, having a disease may impact on one’s own self-conception (ibid, para 4.9). Scully (2017) explains this as the ‘indirect effect’ of MRT in which the application of MRT will affect the child’s life experience (whether with or without certain diseases).

Life experiences that are usually formed by events that happened in life help construct one’s inner stories. It consists of the facts about one’s life and one’s relationship to other individuals, ‘some of which precede numerical existence’ (Malek 2006, p. 91). Somers suggests that the connectivity of past events would affect one’s narrative development. The past events and the connections from them (Somers calls it ‘causal emplotment’) can be used as a mode of explanation as it explains ‘why a narrative has the storyline it does’ (Somers 1994, p. 616). As such, it is arguable that one’s genetic makeup or mode of conception could constitute one of the ‘past events’ in our life and be one of the ‘causal emplotments’ in which it influences the way we relate to our family and the wider society as well as the decisions that we might be making. For instance, the knowledge that one is born via HHGE may influence one’s future reproductive plans given the heritable effects of HHGE. This is in line with the notion that narrative identity has an instrumental value that relates closely to self-creation, where DeGrazia explains it as the deliberate shaping of one’s action or life direction (DeGrazia 2005a,p. 106). This narrative-based identity interest is indeed the basis for the claim for a right to know one’s mode of conception, especially in the context of HHGE (Liaw et al. 2021, pp. 12–13).

Furthermore, having a genetic-related condition or not may also contribute to one’s construction of their stories based on the quality of life and lived experience. One related view is the increasing advocacy in disability studies on more inclusion of people with disabilities or genetic-related conditions as part of the development and evaluation of genome editing technologies for their insights in informing decisions about suitable targets for genome editing (Boardman 2020; Wolbring and Diep 2016). This is also relevant to understanding the broader impacts of HHGE on people with lived experience of genetic conditions as individuals as well as a group (Boardman 2020, p. 126). The latter also reminds us that narrative identity is not only shaped from a first-person perspective but is also influenced by the social context and interactions with others.

This socially mediated identity helps explain how one sees his/her own story as plausible by interpreting how the wider society sees oneself (DeGrazia 2005a, p. 86). In the context of MRT, Scully (2017) also claims that what is important is the social-mediated (narrative) identity in which the focus is on the social and cultural influence (for instance, how MRT is represented in the public media) and how that may impact the mitochondrial-donor-conceived people (Scully 2017). Following this, it is not hard to imagine how the media portrays HHGE and how society reacts to such technology may also influence the individuals born via the procedure as well as existing people who may be affected by the employment of HHGE. The implication of this is that messages that are sent out by the use of HHGE and the social consequence of removing disabling traits using HHGE should be given careful consideration. More inclusion of diverse individuals including people with actual lived experiences should be given further emphasis as part of advocating responsible use of HHGE.

### A multi-faceted concept of identity as a framework in the genome editing debate

The discussion so far suggests that there is indeed a close relationship between genes and identity. If genes were used solely to explain one’s identity, then there is a risk that one is committing what is called the ‘genetic determinism’. This notion has been criticised for being against biological understanding (since our behaviours are also influenced by our external exposure apart from genetic inheritance) (Johnson 2007). It is however not the case that genes in fact *determine* who we are (Resnik and Vorhaus 2006). A multi-faceted notion of identity sees genetic identity as only one part of one’s identity. Although this ‘facet’ can be significant in shaping an individual in terms of some of his/her main features or characteristics, it is not sufficient to define or determine who he/she really is. Therefore, even when much attention has been paid to genetic identity in the discussion of HHGE, concerns about genetic determinism can be avoided if a multi-faceted concept of identity were adopted. This is because a multi-faceted identity also considers other aspects of identity such as the qualitative and narrative identity which are built upon both internal and external factors.

Identity-related considerations can invoke strong reasons against or in favour of deploying technologies such as HHGE. These concerns could and should be addressed and mitigated by policies or good governance. This can be achieved by giving greater attention to public engagement with the cultural norms and values of those who are impacted by technologies properly considered (Almeida and Ranisch 2022; cf Klingler et al. 2022). Despite each notion of identity may contribute varying significance or implications to the HHGE debate, a multi-faceted concept of identity can act as a framework for discussions because it highlights that the notions of identity are non-identical yet might be interrelated to each other.

The interactions of different types of identities can be explained as follows. In the case of HHGE, genetic identity is particularly important (perhaps as has been implied in the debate involving MRT – where the authorities actively claim that the mitochondria do not influence the child’s identity unlike the nuclear genomes, so it is arguably that nuclear genome contributes to child’s identity and influence of one’s identity is of importance). A child born with a genetic disease or not due to the genetic modification done prior to birth may mean a change in the qualitative identity besides the genetic identity. Such a change may in turn be important in one’s narrative identity. A change in genetic identity and knowledge of such may serve as an interpretative and constitutive tool in the child’s developing process. Besides, it also serves as an explanatory tool especially when the body is in an unexplained state (perhaps because of some unforeseen side-effect which has never been discovered before) due to the modification.

As has already been shown, numerical identity is useful to inform the discussion relating to modification done at the stage of gametes and/or embryos as well as the future generations. On the other hand, qualitative, narrative and perhaps social identity may inform the discourse concerning the actual child born via the procedure as well as people living with genetic conditions where HHGE may have an influence on them. The non-identical yet interrelated accounts of identities may together inform the regulatory and ethical implications of the use of HHGE and influence the eventual policy outcome; this may not be achieved if a single layer of identity were to be adopted. For instance, if the discussion is only based on a narrative-based account of identity, it seems that one may easily dismiss it for the case involving embryos on the grounds that embryos do not have the capacity to form any narratives. A multi-faceted concept of identity which highlights that the notions of identity are distinct yet related to each other thus further promoting coherence to the overall discourse on HHGE.

A multi-faceted concept of identity as a framework for discussions also avoids conceptual misconnections and concept creep which may otherwise be ethically problematic. Conceptual misconnections happen when the speaker expresses something with a particular meaning in mind, but this is interpreted as another meaning by the audience, though both of the meanings adopted by the speaker and the audience can be right in their own disciplines (Henschke 2010, p. 436). This is likely to occur when the concept of ‘identity’ is understood as one single facet and when there is a lack of consciousness that there are different aspects to them that ultimately form the whole identity. This is because when the concept of ‘identity’ is taken as a single conception, one may then adopt whatever meaning he deems fit in his own field or whatever meaning is available within his knowledge realm. Even when different conceptions are noted, confusion may still occur if there is no clear assignation of what ‘identity’ means in the context. This is especially true in how HHGE is reported in the popular news media, a common avenue where the public gets informed of a new science such as HHGE – the influence on ‘identity’ is frequently brought up without further explanation. Hence, adopting a multi-faceted concept of identity in the discussion may avoid unnecessary confusion derived from the understanding of ‘identity’ by emphasising the possibility of ‘identity’ being interpreted differently and that they are interconnected with each other.

Apart from the confusion and a lack of clarity, conceptual misconnections can also lead to a scenario called ‘concept creep’ which is arguably ethically problematic. As Henschke (2010, p. 451) explains, concept creep occurs when ‘a word that was initially intended to mean concept A takes on concept B’ and this is ethically problematic especially when one concept is ethically significant, and the other is not. This is particularly relevant in the discussion involving ‘identity’. One of the commonly raised arguments in the debate on the use (or not use) of germline editing (including HHGE) is whether or not genetic modification would affect the ‘identity’ of children (Frankel and Hagen 2011). This may involve different meanings depending on which notion of identity is in mind. Concept creep happens when the word ‘identity’ has been interpreted with another meaning (rather than the claimant’s intended meaning) in an argument used in the ethical debate. This may then direct policy in ways that are problematic.

## Conclusion: summary

The concept of ‘identity’ is no doubt significant in the context of HHGE: how much changes made to the genes or genomes would constitute a change to ‘identity’ and how would an identity change bring socio-ethical implications are questions that cannot be neglected in the related policy and regulations. The concept of ‘identity’ cannot be well-explained without understanding that there are different types of ‘identity’. This paper has contributed to the existing literature by presenting an inclusive analysis of ‘identity’ in the current HHGE debate, including genetic identity, numerical identity, qualitative identity and narrative identity. It is hoped that this could clear the possible confusion caused by a lack of understanding of the various interpretations of ‘identity’. The moral significance of a change in a single aspect of identity by HHGE is insufficient to determine the overall ethical implication of HHGE. In order to have a complete and clear debate, I emphasised that the discussion on HHGE should look at ‘identity’ from these different aspects as a whole. While there are different types of ‘identity’, it is important to note that these concepts are interconnected. For instance, a change in genetic identity due to HHGE intervention may change the qualitative and numerical identity which may further influence one’s narrative and social identity. These observations can be well-captured by a multi-faceted concept of identity I proposed in this paper which consolidates different interpretations of identity from both ontological and empirical perspectives and highlights the *interrelatedness* between the different types of ‘identity’ in the HHGE context. This multi-faceted concept of identity creates a framework that compels the public and stakeholders interested in the employment of HHGE to engage with different kinds of identity constructively and address identity-related issues within this framework.

## Footnotes

1 The term “genetic engineering” (or, “genome engineering”) is usually taken as an umbrella term to mean any intentional manipulation of genes/genomes and thus can cover both somatic and germline genome editing (see for instance, Evans 2002, 1).

2 This can be explained through the concept of genome variations in which it emphasises the differences each of us has in the sequence of DNA. For more details, see Genome News Network 2023.

3 Wrigley, Wilkinson and Appleby in the discussion of mitochondrial replacement technique (MRT) in which they distinguish between the techniques of maternal spindle transfer and pronuclear transfer. They argue that the non-identity problem arises in the application of maternal spindle transfer because the procedure is carried out before fertilisation of the egg, thereby leading to a different sperm fertilising the egg than the one that would have been fertilised without the procedure. In the case of pronuclear transfer, however, the non-identity problem, as it is argued, does not arise because the procedure happens after fertilisation (that is on the *selected embryos*).

4 It is worth noting that this is based on the idea that to be born alive is always good. Nonetheless, this idea is not without any controversy. See for instance, Holtug and Sandoe (1996); Benatar (2006) where Benatar argues that it is possible to be harmed by being brought into existence.

5 In simple terms, epigenetics explains how our genes interact with the molecules within our body and it decides how much or whether some genes are expressed in different cells in our body (Simmons 2008). Epigenetic factors are also influenced by the external environment, including our diet and habits and epigenetic changes may then contribute to how one’s genes are being expressed.

6 There are also more traditional approaches in determining numerical identity, dealing with the persistence question including the psychological approach (see for instance, Locke 1690; Olson 2023; Piccirillo 2010) and the bodily approach (Olson 1997; DeGrazia 2005a; Blatti 2019). These are excluded in the text as they may not be directly applicable to the case of HHGE.

## References

[CR1] Almeida Mara, Ranisch Robert (2022). Beyond safety: Mapping the ethical debate on heritable genome editing interventions. Humanities and Social Sciences Communications.

[CR2] Alonso Marcos, Savulescu Julian (2021). He Jiankui’s gene-editing experiment and the non-identity problem. Bioethics.

[CR3] Benatar, David. 2006. Better never to have been: the harm of coming into existence. Oxford Scholarship.

[CR4] Blatti, Stephan. 2019. Animalism. Stanford Encyclopaedia of Philosophy. Retrieved September 03 2023 from https://plato.stanford.edu/entries/animalism/#OurPer.

[CR5] Boardman Felicity (2020). Human genome editing and the identity politics of genetic disability. Journal of Community Genetics.

[CR6] Boniolo Giovanni, Testa Giuseppe (2012). The identity of living beings, epigenetics, and the modesty of philosophy. Erkenntnis.

[CR7] Bredenoord Annelien (2011). Ethics of modifying the mitochondrial genome. Journal of Medical Ethics.

[CR8] Chadwick Ruth, ten Have Henk, Gordijn Bert (2001). Genetic interventions and personal identity. Bioethics in a European perspective.

[CR9] Cohen, Glenn. 2011. Regulating reproduction: the problem with best interests. Minnesota Law Review. https://ssrn.com/abstract=1955292

[CR10] Beriain deIñigo Miguel (2018). Human dignity and gene editing: using human dignity as an argument against modifying the human genome and germline is a logical fallacy. EMBO reports.

[CR11] Beriain deIñigo Miguel (2020). Gene editing and disabled people: A response to Felicity Boardman. Journal of Community Genetics.

[CR12] DeGrazia David (2005). Enhancement technologies and human identity. Journal of Medicine and Philosophy.

[CR13] DeGrazia David (2005). Human identity and bioethics.

[CR14] Division of the Ethics of Science and Technology of UNESCO. 1999. Birth of the universal declaration on the human genome and human rights. Retrieved September 02 2023 from http://unesdoc.unesco.org/images/0011/001193/119390eo.pdf.

[CR15] Doolabh Keyur (2019). Is the non-identity problem relevant to public health and policy? An online survey. BMC Med Ethics.

[CR16] Evans John (2002). Playing God?.

[CR17] Feeney Oliver, Rakić Vojin (2021). Genome editing and ‘disenhancement’: Considerations on issues of non-identity and genetic pluralism. Humanities and Social Sciences Communications.

[CR18] Feinberg Joel (1986). Wrongful life and the counterfactual element in harming. Social Philosophy & Policy.

[CR19] Frankel, Mark S and Brent T Hagen. 2011. Background paper for Nuffield Council on Bioethics: germline therapies. Retrieved September 02 2023 from https://www.nuffieldbioethics.org/assets/images/Germline_therapies_background_paper.pdf

[CR20] Garnham, Thomas. 2016. Human beings can now be edited, but we may not like the results (*The Scotsman October 25,* 2016) Retrieved September 04 2023 from https://www.scotsman.com/news/opinion/columnists/human-beings-can-now-be-edited-but-we-may-not-like-the-results-1465071

[CR21] Genome News Network. 2023. Genome variations. Retrieved September 02 2023 from https://www.genome.gov/about-genomics/educational-resources/fact-sheets/human-genomic-variation.

[CR22] Glannon Walter (2001). Genes and future people: Philosophical issues in human genetics.

[CR23] Goekoop Floor M (2020). Systematic scoping review of the concept of genetic identity and its relevance for germline modification. PLoS ONE.

[CR24] Green Ronald (1997). Parental autonomy and the obligation not to harm the child genetically. Journal of Law, Medicine and Ethics..

[CR25] Griffiths Jack Owen, Ferrarello Susi (2021). Bioethics, the ontology of life, and the hermeneutics of biology. Phenomenology of bioethics: technoethics and lived-experience.

[CR26] Harris John (2000). The welfare of the child. Health Care Analysis.

[CR27] Hauskeller Christine (2004). Genes, genomes and identity projections on matter. New Genetics and Society.

[CR28] Henschke Adam (2010). Did you just say what I think you said? talking about genes, identity and information. Identity in the Information Society.

[CR29] Holm Soren (2019). Let us assume that gene editing is safe – the role of safety arguments in the gene editing debate. Cambridge Quarterly of Healthcare Ethics.

[CR30] Holtug, N., Peter S., 1996 Who benefits? Why personal identity does not matter in a moral evaluation of germ-line gene therapy. *Journal of Applied Philosophy*. 10.1111/j.1468-5930.1996.tb00158.x

[CR31] Johnson Wendy (2007). Genetic and environmental influences on behaviour: Capturing all the interplay. Psychological Review.

[CR32] Juth Niklas (2016). Germline genetic modification, CRISPR, and human identity: Can genetics turn you into someone else?. Ethics, Medicine and Public Health..

[CR33] Klingler Corinna (2022). Public engagement with brain organoid research and application: Lessons from genome editing. AJOB Neuroscience.

[CR34] Liao Matthew (2017). Do mitochondrial replacement techniques affect qualitative or numerical identity?. Bioethics.

[CR35] Liaw Ying-Qi, Turkmendag Ilke, Hollingsworth Kathryn (2021). Reinterpreting “genetic identity” in the regulatory and ethical context of heritable genome editing. New Genetics and Society.

[CR36] Locke, John. 1690. An essay concerning human understanding. Retrieved September 03 2023 from https://www.gutenberg.org/files/10615/10615-h/10615-h.htm.

[CR37] Mackie, Penelope and Mark Jago. 2022. Transworld identity. *Stanford Encyclopaedia of Philosophy*. Retrieved September 03 2023 from https://plato.stanford.edu/entries/identity-transworld/.

[CR38] Malek Janet (2006). Identity, harm and the ethics of reproductive technology. Journal of Medicine and Philosophy.

[CR39] Martin Paul (2020). Genome editing: The dynamics of continuity, convergence, and change in the engineering of life. New Genetics and Society.

[CR40] National Academies of Sciences, Engineering, and Medicine, NASEM. 2017. *Human genome editing: science, ethics and governance*. The National Academic Press. 28796468

[CR41] Noonan, Harold and Ben Curtis. 2022. Identity. *Stanford Encyclopaedia of Philosophy*. Retrieved September 03 2023 from https://plato.stanford.edu/entries/identity/.

[CR42] Nuffield Council on Bioethics, Nuffield. 2012. Novel techniques for the prevention of mitochondrial DNA disorders: an ethical review. Retrieved September 03 2023 from https://www.nuffieldbioethics.org/assets/pdfs/Novel_techniques_for_the_prevention_of_mitochondrial_DNA_disorders.pdf.

[CR43] Olson Eric (1997). The human animal: Personal identity without psychology.

[CR44] Olson, Eric. 2023. Personal identity. *Stanford Encyclopaedia of Philosophy*. Retrieved September 03 2023 from https://plato.stanford.edu/entries/identity-personal/#UndPerQue

[CR45] Omerbasic Alina, Braun Matthias, Schickl Hannah, Dabrock Peter (2018). Genome editing, non-identity and the notion of harm. Between moral hazard and legal uncertainty 67–81.

[CR46] Parfit Derek (1984). Reasons and persons.

[CR47] Parker, Michael. 2005. The welfare of the child. Human Fertility (Cambridge, England). 10.1080/14647270500050371 10.1080/14647270500050371 15823846

[CR48] Petersen Alan (2006). The best experts: The narratives of those who have a genetic condition. Social Science & Medicine.

[CR49] Piccirillo, Ryan A. 2010. The Lockean memory theory of personal identity: definition, objection, response. *Inquiries Journal*. Retrieved September 02 2023 http://www.inquiriesjournal.com/a?id=1683.

[CR50] Postan, Emily. 2017. Defining ourselves: narrative identity and access to personal biological information. PhD Article, The University of Edinburgh. Retrieved September 02 2023 from https://era.ed.ac.uk/bitstream/handle/1842/25733/Postan2017.pdf?sequence=1&isAllowed=y

[CR51] Resnik David, Vorhaus Daniel B (2006). Genetic modification and genetic determinism. Philosophy, Ethics and Humanities in Medicine..

[CR52] Ribeiro Barbara (2018). Introducing the dilemma of societal alignment for inclusive and responsible research and innovation. Journal of Responsible Innovation.

[CR53] Rosenberg, Leon and Diane Drobnis Rosenberg. 2012. Human genes and genomes. Elsevier.

[CR54] Rulli Tina (2019). Reproductive CRISPR does not cure disease. Bioethics.

[CR55] Salvi Maurizio (2001). Shaping individuality: Human inheritable germ line gene modification. Theoretical Medicine.

[CR56] Scully Jackie Leach (2017). A mitochondrial story: Mitochondrial replacement, identity and narrative. Bioethics.

[CR57] Shekhtman, Lonnie. 2015. Science's 'Breakthrough of the Year' inspired as much trepidation as hope (*The Christian Science Monitor* December 18, 2015) Retrieved September 04 2023 https://www.csmonitor.com/Science/2015/1218/Science-s-Breakthrough-of-the-Year-inspired-as-much-trepidation-as-hope

[CR58] Sholtis Samuel, Noonan James (2010). Gene regulation and the origins of human biological uniqueness. Trends in Genetics.

[CR59] Simmons, Danielle. 2008. Epigenetic influences and disease. *Nature Education*. Retrieved September 03 2023 https://www.nature.com/scitable/topicpage/epigenetic-influences-and-disease-895/.

[CR60] Sollberger, Daniel. 2013. On identity: from a philosophical point of view. *Child & Adolescent Psychiatry & Mental Health*. Retrieved September 02 2023 from https://psycnet.apa.org/record/2014-46026-001 10.1186/1753-2000-7-29 PMC3751052 23902741

[CR61] Somers MR (1994). The narrative constitution of identity: A relational and network approach. Theory and Society.

[CR62] Steering Committee on Bioethics. 2000. Preparatory work on the convention on human rights and biomedicine. Retrieved March 03 2023 from https://www.coe.int/t/dg3/healthbioethic/texts_and_documents/CDBI-INF(2000)1PrepConv.pdf.

[CR63] van Beers Britta C (2020). Rewriting the human genome, rewriting human rights law? Human rights, human dignity, and human germline modification in the CRISPR era. Journal of Law and the Biosciences.

[CR64] Varki Ajit, Geschwind Daniel H, Eichler Evan E (2008). Explaining human uniqueness: Genome interactions with environment behaviour and culture. Nature Reviews Genetics.

[CR65] Wolbring Gregor, Diep Lucy (2016). The discussions around precision genetic engineering: Role of and impact on disabled people. Laws.

[CR66] Wrigley Anthony, Wilkinson Stephen, Appleby John (2015). Mitochondrial replacement: Ethics and identity. Bioethics.

[CR67] Zeiler Kristin (2007). Who am I? When do “I” become another? An analytic exploration of identities, sameness and difference, genes and genomes. Health Care Analysis.

[CR68] Zuradzki, Tomasz. 2008. Genetic engineering and the non-identity problem. *Diametros*. Retrieved September 02 2023 from https://philarchive.org/archive/URAGEA.

